# Filtering Gene Ontology semantic similarity for identifying protein complexes in large protein interaction networks

**DOI:** 10.1186/1477-5956-10-S1-S18

**Published:** 2012-06-21

**Authors:** Jian Wang, Dong Xie, Hongfei Lin, Zhihao Yang, Yijia Zhang

**Affiliations:** 1 School of Computer Science and Technology, Dalian University of Technology, Dalian, China

## Abstract

**Background:**

Many biological processes recognize in particular the importance of protein complexes, and various computational approaches have been developed to identify complexes from protein-protein interaction (PPI) networks. However, high false-positive rate of PPIs leads to challenging identification.

**Results:**

A protein semantic similarity measure is proposed in this study, based on the ontology structure of Gene Ontology (GO) terms and GO annotations to estimate the reliability of interactions in PPI networks. Interaction pairs with low GO semantic similarity are removed from the network as unreliable interactions. Then, a cluster-expanding algorithm is used to detect complexes with core-attachment structure on filtered network. Our method is applied to three different yeast PPI networks. The effectiveness of our method is examined on two benchmark complex datasets. Experimental results show that our method performed better than other state-of-the-art approaches in most evaluation metrics.

**Conclusions:**

The method detects protein complexes from large scale PPI networks by filtering GO semantic similarity. Removing interactions with low GO similarity significantly improves the performance of complex identification. The expanding strategy is also effective to identify attachment proteins of complexes.

## Background

Protein complexes are important molecular entities in cellular organizations. With large amounts of protein interactions produced by high-throughput experimental techniques [1,2], protein complexes are able to be automatically identified from genome-scale interaction networks by computational approaches. Generally, proteins in a complex share more interactions among themselves than with other proteins [3]. Many algorithms, based on graph theory, have been proposed to identify protein complexes by detecting dense regions in PPI networks, such as MCODE [4], MCL [5], and CFinder [6]. However, their performance is affected by the false-positive interactions in the network. In some experiments, the proportion of false-positive interactions generated by high-throughput techniques is estimated to be up to 50% [7]. It is reasonable to make use of biological information to measure the reliability of interaction pairs or predicted complexes. For example, protein function annotation datasets are used in RNSC [8] and DECAFF [9] to filter complexes with low functional homogeneity or reliability.

GO annotation is a useful information resource to measure the reliability of protein interaction pairs. The GO project maintains three structured controlled vocabularies, which describe gene products in terms of their associated biological processes, cellular components, and molecular functions [10]. The ontology of each domain is structured as a directed acyclic graph (DAG), which organizes terms by their relationships. The similarity of two gene products based on GO annotations can be considered as the similarity of two sets of GO terms. The semantic similarity of GO terms can be measured by the topological information in the ontology structure.

In this paper, we attempt to make use of GO annotations and the ontology structure of GO terms to measure semantic similarity of GO terms and proteins. The similarity of two GO terms is measured based on their average distance to their lowest common ancestors in the ontology structure. Semantic similarity between proteins is computed as the similarity of two sets of GO terms, which annotate the two proteins respectively. PPIs in the network are then weighted by the similarity of interacting proteins for the filtering and clustering steps. As far as we know, most approaches filter the predicted complexes with low density or statistical significance in post processes [4,9,11,12], which still introduce some unreliable interactions in the results. In our method, however, the low-weight interactions are filtered first, followed by a cluster-expanding algorithm to identify high quality complexes consisting of only reliable interactions. Considering the core-attachment structure revealed by Gavin et al. [13], which reflects the inherent organization of protein complexes, we propose a network clustering algorithm to identify the core and attachment proteins of complexes successively. Firstly, cliques in the filtered network are detected. Highly overlapping cliques are merged to form cores of complexes. Secondly, we add attachment proteins to the cores, making use of the cluster-expanding strategy in RRW algorithm [11], which is appropriate for expanding clusters consisting of multiple nodes in weighted networks. By applying the clustering algorithm on the purified PPI network, our method identifies complexes with high biological significance and functional homogeneity.

## Methods

In this section, we present, in detail, the two phases used in our approach. In the first phase, protein semantic similarity is computed based on their GO annotations. Following this, a core-attachment structure detection algorithm is applied to detect core and attachment proteins of complexes from the filtered PPI network. The flow of our method can be described in the following steps:

(1) Computing protein semantic similarity for every pair of proteins with interaction in the PPI network.

(2) Removing interactions with low similarity from the original network.

(3) Finding cliques in the filtered network to form complex cores. Multiple highly overlapping cliques are merged to form one core.

(4) Adding attachment proteins to these cores with the expanding strategy in RRW algorithm.

### Semantic similarity for PPI

The GO database is currently one of the most comprehensive and well-curated ontology databases in the bioinformatics community. The ontology structure of GO terms is organized as DAGs of three domains with terms as nodes and their relationships as directed edges. The GO terms are structured by two kinds of relationships to each other: "is-a" and "part-of", representing specific-to-general and part-to-whole relations respectively.

Semantic similarity of GO terms can be measured by their positions in the DAGs. In the task of semantic similarity computation, we attempted to design our GO semantic similarity measure based on a graph-based method measuring concepts in a taxonomy structure [14]. In the ontology structure, the semantic specificity of a given term *x *can be measured by the path length from the root node to *x *passing through its ancestors. In a similar way, given a term *x*, its relative semantic specificity from its ancestor *a *can be measured by the path length from *a *to *x*. Since there may be multiple paths from one node to another in DAGs, we define distance *d*(*a*, *x*) as the average path length from term *a *to *x*, while *a *is one of ancestors of *x*. Two terms, *x *and *y*, are considered more similar if their distances to their lowest common ancestors are shorter, or their lowest common ancestors average distance to the root is longer. We define *LCA*(*x*, *y*) as the set of lowest common ancestors of term *x *and term *y*. For the node set of common ancestors of × and y, *a *∈ *LCA*(x, y) if the paths from *a *to *x *and *a *to *y *do not pass through any other common ancestor. Based on the graph characteristics of GO terms, we define the similarity of two GO terms *x *and *y Sim*(*x*, *y*) as follows:

(1) $$Sim\left(x,y\right)=\frac{\underset{a\in LCA\left(x,y\right)}{\sum }\frac{d{\left(root,a\right)}^{2}}{d_{a}\left(root,x\right)d_{a}\left(root,y\right)}}{\left|LCA\left(x,y\right)\right|}$$

where *root *denotes a virtual node as the parent node of the three root nodes of three distinct DAGs (biological process, cellular component and molecular function) in GO. *d_a_*(*root*, *x*) denotes the average length of paths from *root *to *x *passing through *a*, *d_a_*(*root*, *x*)=*d*(*root*, *a*)+*d*(*a*, *x*). *Sim*(*x*, *y*) reaches its minimum value zero when *x *and *y *are terms in different domains, while it reaches its maximum value 1 when *x *and *y *are the same term.

By the definition of term-wise similarity, we can measure the similarity of two proteins annotated by two sets of GO terms. We calculate each pair of GO terms in annotation sets of two proteins, and use the best-match average approach [15] to evaluate the overall similarity of the two term sets:

(2) $$PSim\left(A,B\right)=\frac{\underset{x\in T_{A}}{\sum }{\text{max}}_{y\in T_{B}}\left(sim\left(x,y\right)\right)+\underset{y\in T_{B}}{\sum }{\text{max}}_{x\in T_{A}}\left(sim\left(x,y\right)\right)}{\left|T_{A}|+|T_{B}\right|}$$

*T_A _*and *T_B _*denote the term sets annotating protein *A *and *B *respectively. For every term *x *in *T_A_*, we find the most similar terms in *T_B _*to calculate $\underset{y\in T_{B}}{\text{max}}\left(sim\left(x,y\right)\right)$, and vice versa. Then we consider the average value of these term-pair similarity values as the similarity of protein *A *and *B*, which is also a uniform result.

### Network clustering

We use *PSim *similarity to weight every pair-wise interaction in the PPI network. Considering the inaccuracy of interaction network, we remove the interactions with a *PSim *value no larger than a threshold *filter_thres*. Only high quality interactions are involved in the following complex identification steps.

The core-attachment structure [13] provides an insight view of inherent organization of protein complexes. Several methods such as COACH [16] and CORE [17] have made good use of this characteristic to detect protein complexes from PPI networks. The core proteins of a complex have relatively more interactions among themselves and share a high degree of functional similarity. Attachment proteins are the surrounding proteins of the core performing relative functions.

In our algorithm, we first used the clique finding algorithm as described in [18] to identify all cliques in the network. Then, highly overlapping cliques are merged to form larger clusters if their neighborhood affinity *NA *defined as follows is above threshold *merge_thres*:

(3) $$NA\left(A,B\right)=\frac{|V_{A}\cap V_{B}{|}^{2}}{\left|V_{A}|\times |V_{B}\right|}$$

where *V_A _*and *V_B _*denote the node sets of clique *A *and *B *respectively. All of the merged clusters and cliques not involved in the merging form core set of the complex. Attachment proteins are added to each core by the expanding strategy of RRW algorithm [11]. RRW is an appropriate algorithm for cluster expanding as it simulates a random walk with a restart probability starting from multiple nodes in a network. After computing the stationary vector of every single node in network, the RRW algorithm expands clusters starting from every node, adding one node to the cluster and saving the expanded cluster in each expanding step. Then, the clusters are sorted and filtered by their statistical significance. Since this filtering strategy tends to generate relatively small sized clusters, we use the expanding strategy to run repeated random walk from every core protein set with neighbor nodes, and only add the maximal expansion of each cluster to the result set. The original parameters of the minimum and maximum cluster size of RRW are 5 and 11, while the size distributions of hand-curated complexes from CYC2008 [19], Aloy [20] and MIPS [21] indicate that most complexes are of a size between 2 and 20. We set the parameters to 2 and 20 respectively in our method while other parameters are set to default.

**Figure 1:**
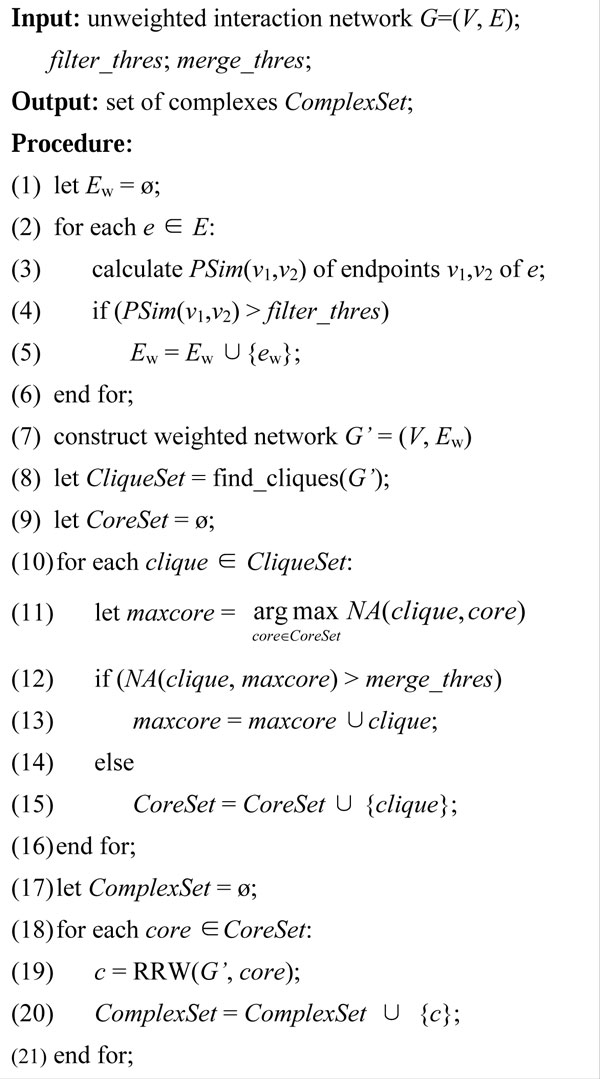
**Flow of our algorithm**.

The flow of our algorithm is described by pseudo-codes in Figure 1. The computation of protein semantic similarity is executed in step (1) to (6), in which *E_w _*denotes the weighted edge set. After construction of the weighted network *G'*, cliques are detected by algorithm [18] in step (8). The procedure of a clique merging is described in step (10) to (16). In step (19) RRW(*G'*, *core*) denotes the RRW expanding procedure starting from a cluster core. RRW(*G'*, *core*) computes affinity score between each protein to the given cluster based on the random walk stationary vectors generated from *G'*. The closet protein to the cluster is added to the cluster in each expanding step. This process is continued until no protein's affinity score reaches a given threshold. We collect only the maximal expansion of each cluster as a predicted complex, which is different from the original RRW algorithm.

## Results

We apply our algorithm on three datasets of yeast protein interactions: Gavin [13], Krogan [22], and DIP [23]. The details of the interaction datasets are shown in Table 1. Two complex datasets are used as benchmark for evaluation. One is CYC2008 [19] with 408 complexes used as benchmark complexes in most approaches. The other one, named as "Combined" below, is the union of Aloy[20], MIPS[21], and SGD database[24] with 426 complexes used in COACH [16], [25]and [26].

**Table 1:** Details of interaction datasets

Datasets	Number of proteins	Number of interactions
Gavin	1430	6531
Krogan	3581	14076
DIP	4928	17201

The GO resource we used can be downloaded from http://www.geneontology.org/ with version 1.2028, dated 06/10/2011. The version of the annotation file of Saccharomyces cerevisiae is 1.1566 submitted on 06/18/2011.

We evaluate the experimental result with six evaluation metrics: precision (P), recall (R), F-measure (F), sensitivity (Sn), PPV and accuracy (Acc), which are described in [26]. A predicted complex is matched with a benchmark complex if their *NA *is above 0.2, which is used in most approaches.

### Parameter selection

**Figure 2:**
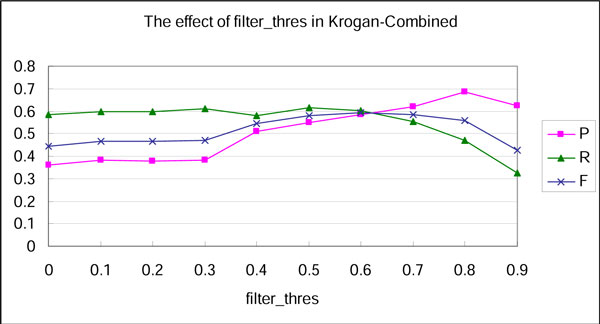
**The effect of filter_thres**.

Before comparing with other approaches, the influence of parameters was examined in our method. To optimize our method, the edge filtering threshold, i.e., *filter_thres*, was set from 0 to 0.9 by an increment of 0.1 each time. To observe how *filter_thres *affected the result, the *merge_thres *was fixed to 1, which led to unavailable merging step. The precision, recall, and F-measure with Krogan-Combined datasets influenced by different *filter_thres *are shown in Figure 2.

With the increase of *filter_thres*, the precision rises in general, indicating that high accurate complexes can be identified from high quality interactions. Therefore, removing interaction pairs with low similarity significantly improves the performance of complex identification. The GO semantic similarity measure we proposed is effective in estimating the quality of PPI. The F-measure reaches maximum when *filter_thres *is set to an optimal value 0.6, which is also validated by other combinations of network and benchmark datasets. In addition, we found that the number of predicted complexes is inversely proportional to *filter_thres*. This number is above 1,000 when *filter_thres *is less than 0.3, which seems unreasonable for a network with 3581 nodes. This is because the clique finding algorithm [18] generates cliques starting from every nodes in network. Many of these cliques have a high proportion of common nodes. It is necessary to merge the large amounts of overlapping cliques.

**Figure 3:**
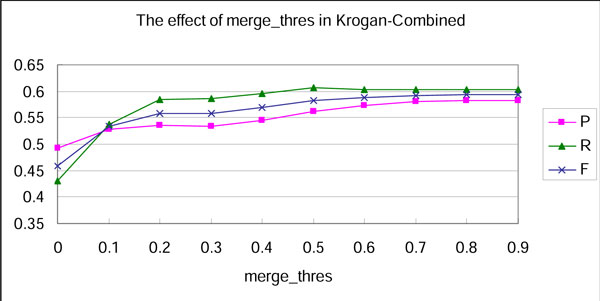
**The effect of merge_thres**.

We present another experiment to find optimal *merge_thres*. As shown in Figure 3, the best result is generated by stepping over the merging step as *merge_thres *set to 1. However, the F-measure is improved solely with the increase of precision, while recall keeps the same value when *merge_thres *changes from 0.5 to 1. This indicates that the overlapping cliques may introduce matching between multiple similar clusters and a single benchmark complex. According to the definition of precision [26], redundant correct answers in predicted complex set may leads to increase of precision. For a fair comparison with other approaches, we set 0.5 as the optimal value of *merge_thres*.

### Comparison with other approaches

**Figure 4:**
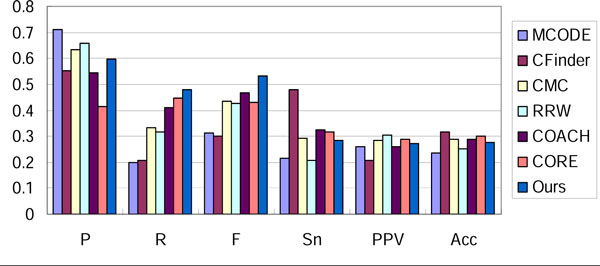
**Performance comparison of various approaches on Gavin-Combined**.

**Figure 5:**
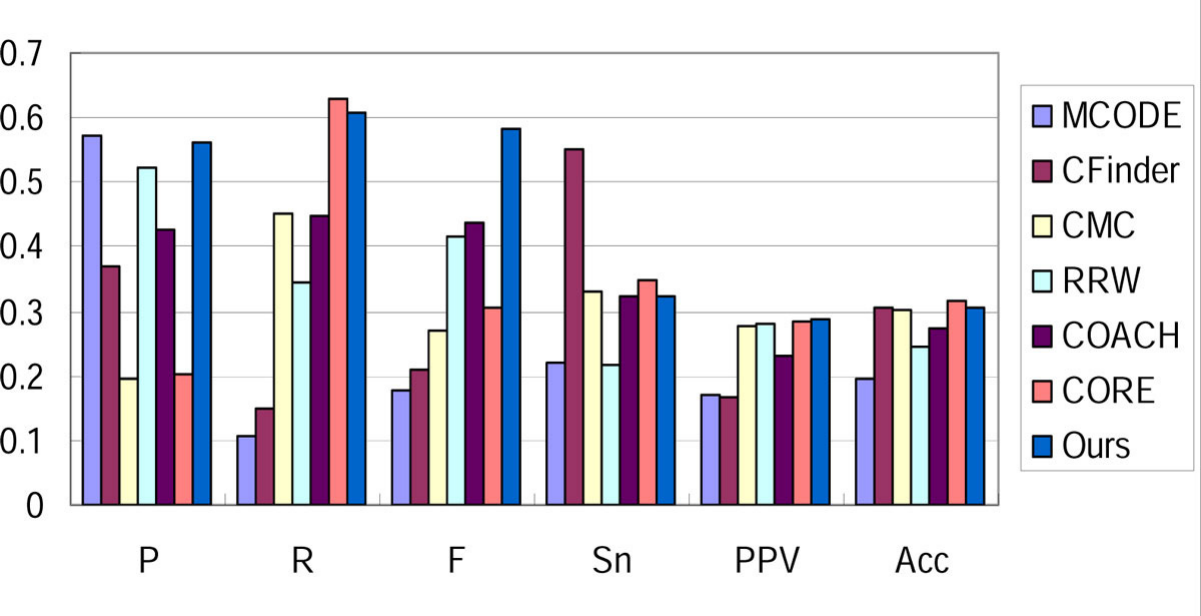
**Performance comparison of various approaches on Krogan-Combined**.

We compared our method with six well-known approaches: MCODE [4], CFinder [6], CMC [12], RRW [11], COACH [16] and CORE [17] with optimal parameters. The result in three networks evaluated with Combined benchmark dataset is shown in Figure 4, 5, 6. Our method outperforms other approaches in the overall evaluation metric F-measure. In the three networks, our method reaches the precision level of MCODE and RRW, while it achieves a higher recall. This implies that noisy interactions preclude the predicted complexes from matching real complexes. These interactions are removed effectively by our filtering steps.

**Figure 6:**
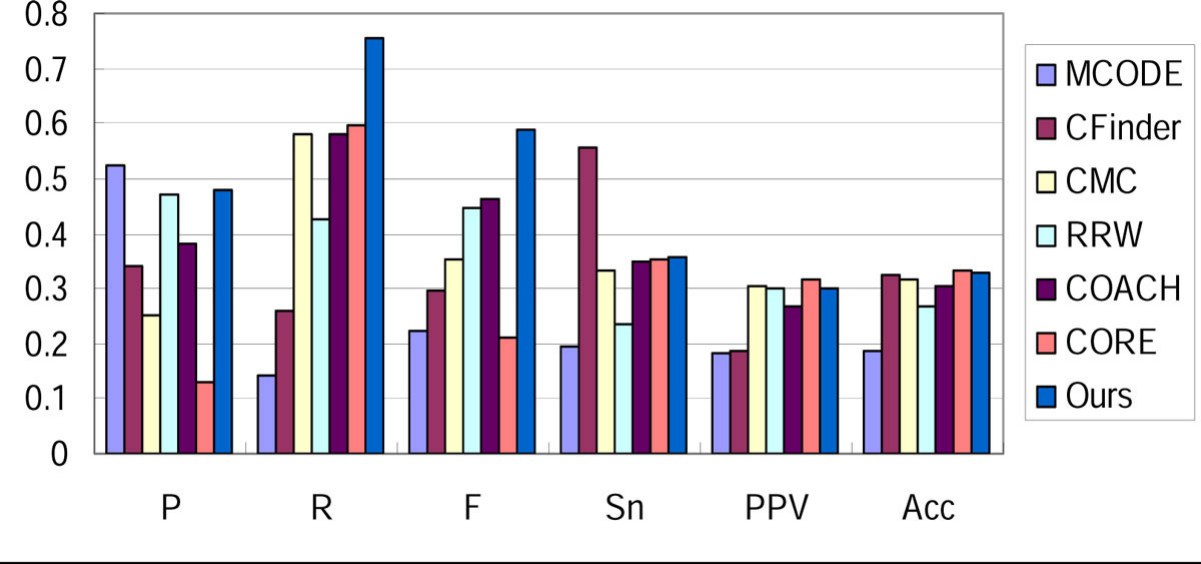
**Performance comparison of various approaches on DIP-Combined**.

Sensitivity, PPV and accuracy are metrics evaluating the correspondence between the prediction and benchmark in micro level. Sensitivity represents the coverage of a complex by its best-matching cluster (the maximal fraction of proteins in the complex found in a common cluster), while PPV measures how well a given cluster predicts its best-matching complex [26]. Accuracy is the geometric average of sensitivity and PPV. By reaching an average level of these evaluation metrics, our method can generate complexes matching more real complexes accurately.

**Figure 7:**
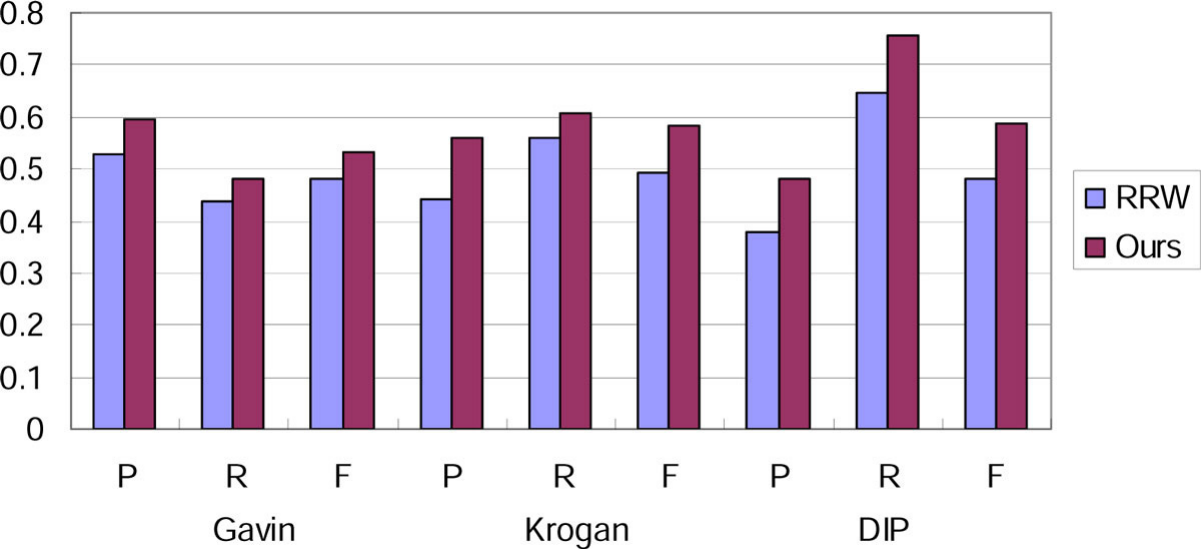
**The effect of core-attachment clustering strategy on filtered networks**.

In Table 2, 3, 4, we demonstrate the comparison results evaluated with CYC2008 benchmark dataset. It is indicated that the performance of our method is similar with different benchmarks. By focusing on the interactions with high GO semantic similarity in the networks, our method achieves higher recall and F-measure than the other approaches. To evaluate the effectiveness of the core-attachment based clustering steps in our algorithm, we compared our method with original RRW algorithm on the same filtered network by the filtering step in our method with *filter_thres *set to 0.6. The parameters of minimum and maximum size in the original RRW algorithm are also set to 2 and 20 respectively. Figure 7 shows the comparison result on filtered networks evaluated by Combined benchmark. It is shown that the design of core-attachment clustering steps is relatively more consistent with real complex structures.

**Table 2:** Performance comparison of various approaches on Gavin-CYC2008

Method	P	R	F	Sn	PPV	Acc
MCODE	0.739	0.154	0.255	0.283	0.519	0.384
CFinder	0.663	0.191	0.297	0.513	0.343	0.419
CMC	0.608	0.218	0.321	0.371	0.606	0.474
RRW	0.704	0.238	0.355	0.294	0.657	0.439
COACH	0.525	0.331	0.406	0.44	0.547	0.49
CORE	0.469	0.38	0.42	0.446	0.585	0.511
Ours	0.678	0.404	0.507	0.405	0.663	0.518

**Table 3:** Performance comparison of various approaches on Krogan-CYC2008

Method	P	R	F	Sn	PPV	Acc
MCODE	0.612	0.081	0.142	0.273	0.345	0.307
CFinder	0.451	0.15	0.225	0.56	0.22	0.351
CMC	0.224	0.377	0.281	0.472	0.58	0.523
RRW	0.581	0.277	0.375	0.32	0.605	0.44
COACH	0.472	0.38	0.421	0.477	0.498	0.487
CORE	0.275	0.691	0.394	0.566	0.537	0.551
Ours	0.626	0.559	0.59	0.509	0.663	0.581

**Table 4:** Performance comparison of various approaches on DIP-CYC2008

Method	P	R	F	Sn	PPV	Acc
MCODE	0.576	0.096	0.164	0.282	0.328	0.304
CFinder	0.396	0.257	0.312	0.612	0.297	0.437
CMC	0.283	0.505	0.363	0.523	0.526	0.525
RRW	0.541	0.365	0.436	0.378	0.557	0.459
COACH	0.418	0.529	0.467	0.545	0.481	0.512
CORE	0.16	0.662	0.258	0.569	0.567	0.568
Ours	0.537	0.699	0.607	0.583	0.578	0.581

### Examples of predicted complexes

**Figure 8:**
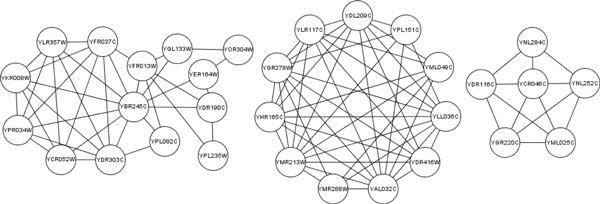
**Topology of complex number 6, 7 and 8 in Table 5**.

The predicted complexes of our approach are generated from high similarity interactions in networks. Therefore, they have high similarity in GO annotations. We present several examples of predicted complexes generated from Gavin dataset in Table 5 with their p-values of the three GO domains. The p-value is the statistical significance of the occurrence of a complex with respect to a GO annotation. Usually a complex is considered to be statistically significant if the p-value is less than 0.01. the p-values of complexes are calculated with Bonferroni correction using the tool SGD's GO::TermFinder [27]. The *NA *scores with their matching real complexes are also listed. As is shown in Table 5, five of them have high matching rates and p-values, while three of them are not matching any complex in two benchmark datasets. The topology of the three complexes is presented in Figure 8. According to their p-values of GO annotations, they have high functional homogeneity. They are possibly potential real protein complexes that have not yet been discovered. These predicted complexes provide clues for biologists to discover new complexes.

**Table 5:** Examples of predicted complexes

ID	predicted complex	NA	GO biological processes		GO molecular functions		GO cellular components	
			
			annotation	p-value	annotation	p-value	annotation	p-value
1	YGR095C YDL111C YGR158C YCR035C YOL142W YHR069C YOR001W YHR081W YDR280W YNL232W YOL021C YGR195W	1	GO:0071051	1.10e-33	GO:0000175	2.77e-19	GO:0000176	1.25e-33
2	YPL243W YML105C YKL122C YPR088C YDL092W YPL210C	1	GO:0006617	3.10e-18	GO:0008312	1.37e-15	GO:0005786	5.59e-19
3	YBR060C YPR162C YNL261W YHR118C YML065W YLL004W	1	GO:0006267	3.07e-15	GO:0003688	3.54e-14	GO:0000808	1.65e-19
4	YHL025W YJL176C YNR023W YOR290C YFL049W YPR034W YBR289W YMR033W YPL129W YDR073W YPL016W	0.917	GO:0042766	5.97e-30	GO:0008094	1.09e-3	GO:0016514	4.90e-33
5	YLR071C YGR104C YOR174W YER022W YOL135C YHR041C YGL025C YDR443C YBR253W YNL236W YHR058C YOL051W YMR112C YNR010W YBR193C YPR070W YPR168W YCR081W YDR308C	0.76	GO:0006366	1.80e-22	GO:0001104	8.82e-54	GO:0016592	4.61e-51
6	YLR357W YFR037C YPR034W YBR245C YFR013W YPL235W YOR304W YDR190C YCR052W YKR008W YGL133W YDR303C YER164W YPL082C	-	GO:0006338	7.87e-24	GO:0016887	9.77e-17	GO:0016585	2.54e-19
7	YDR416W YAL032C YMR288W YMR213W YHR165C YGR278W YLR117C YDL209C YPL151C YML049C YLL036C	-	GO:0000398	2.35e-19	GO:0000384	2.04e-14	GO:0005681	5.52e-21
8	YNL252C YML025C YDR116C YNL284C YGR220C YCR046C	-	GO:0032543	2.74e-7	GO:0003735	2.16e-9	GO:0000315	1.05e-12

## Conclusions

Computational approaches for protein complex detection are often affected by false-positive interactions in large scale PPI data. In this paper, we identify protein complexes in PPI networks with a two-phase method. We first measure the semantic similarity of GO terms and proteins by the ontology structure to evaluate the reliability of PPIs. After removing unreliable proportion of interactions, a core-attachment based clustering method is applied to the filtered network for complex identification. The main contributions of this paper are: 1) proposing a graph-based GO semantic similarity measure to purify the PPI network, 2) designing a core-attachment detection algorithm making use of the RRW algorithm to detect complexes from the filtered network.

By comparing with various approaches, our method outperforms the other approaches in overall evaluations. The graph-based similarity measure enhances the complex identification performance. Removing unreliable interactions before clustering improves the performance significantly. The strategy of expanding clusters by RRW algorithm is also effective to identify the attachment proteins in protein complexes. A future research can focus on the similarity measure of PPI in the network. Various measuring method can be applied to estimate the reliability of protein pairs to filter the false-positive interactions.

## Competing interests

The authors declare that they have no competing interests.

## Authors' contributions

JW carried out the identification of protein complexes studies, participated in the design of the experiments and helped to draft the manuscript. DX carried out the GO database studies, proposed the method of computing protein semantic similarity and draft the manuscript. HL guided the design of the study and participated in the experimental results analysis. ZY participated in the study of PPI netwoks and helped to revise the manuscript. YZ participated in the study of RRW algorithm, and performed the statistical analysis. All authors read and approved the final manuscript.
